# Genome Survey Sequencing of In Vivo Mother Plant and In Vitro Plantlets of *Mikania cordata*

**DOI:** 10.3390/plants9121665

**Published:** 2020-11-27

**Authors:** Yongfeng Hong, Xia Huang, Chunmei Li, Xiaoxian Ruan, Zhen Wang, Yingjuan Su, Ting Wang

**Affiliations:** 1 School of Life Sciences, Sun Yat-sen University, Guangzhou 510275, China; hongyf3@mail2.sysu.edu.cn (Y.H.); huangxia@mail.sysu.edu.cn (X.H.); lichm3@mail.sysu.edu.cn (C.L.); ruanxx@mail2.sysu.edu.cn (X.R.); wangzh535@mail2.sysu.edu.cn (Z.W.); 2 Research Institute of Sun Yat-Sen University in Shenzhen, Shenzhen 518057, China; 3 College of Life Sciences, South China Agricultural University, Guangzhou 510642, China

**Keywords:** *Mikania cordata*, genome survey sequencing, micropropagation, microbial contaminants, SSRs

## Abstract

*Mikania cordata*, the only native congener of the invasive weed *Mikania micrantha* in China, is an ideal species for comparative study to reveal the invasion mechanism. However, its genome resources are lagging far behind its congener, which limits the comparative genomic analysis. Our goal is to characterize the genome of *M. cordata* by next-generation sequencing and propose a scheme for long-read genome sequencing. Previous studies have shown that the genomic resources of the host plant would be affected by the endophytic microbial DNA. An aseptic sample of *M. cordata* will ensure the proper genome in downstream analysis. Because endophytes are ubiquitous in the greenhouse-grown *M. cordata*, the in vitro culture with cefotaxime or timentin treatment was undertaken to obtain the aseptic plantlets. The in vivo mother plant and in vitro plantlets were used to survey the genome. The microbial contamination in *M. cordata* was recognized by blast search and eliminated from the raw reads. The decontaminated sequencing reads were used to predict the genome size, heterozygosity, and repetitive rate. The in vivo plant was so contaminated that microbes occupied substantial sequencing resources and misled the scaffold assembly. Compared with cefotaxime, treatment with timentin performed better in cultivating robust in vitro plantlets. The survey result from the in vitro plantlets was more accurate due to low levels of contamination. The genome size was estimated to be 1.80 Gb with 0.50% heterozygosity and 78.35% repetitive rate. Additionally, 289,831 SSRs were identified in the genome. The genome is heavily contaminated and repetitive; therefore, the in vitro culture technique and long-read sequencing technology are recommended to generate a high-quality and highly contiguous genome.

## 1. Introduction

*Mikania cordata* (Burm. f.) B.L. Robinson, the only congener of the notorious invasive weed *Mikania micrantha* H.B. Kunth in China, is an herbaceous stem-twiner native to tropical Asia [1]. *Mikania cordata* is distributed restrictively in Southeast Asia and South China including Hainan, Taiwan, and southeast Yunnan [1]. It is used as an ethnomedicinal plant in the treatment of cuts and wounds [2]. *Mikania* comprising ca. 450 species is the largest genus in the tribe Eupatorieae of the Asteraceae family [1]. There are only two species of the genus *Mikania* in China: a native species *M. cordata* and an introduced species *M. micrantha* [1]. Both are perennial vines with slender stems, heart-shaped leaves, and pappus-bearing achenes [1,3]. Their appearance so closely resembles each other that it is hard to distinguish *M. cordata* from *M. micrantha* [4,5].

*Mikania micrantha*, also known as “mile-a-minute” weed, is native to tropical America and has spread through Asia and the South Pacific [6]. The creeping and climbing habits enable *M. micrantha* to smother the host plants by penetrating crowns and blocking sunlight [7]. *Mikania micrantha*, as one of the top 100 worst invasive species, has caused severe damage to natural ecosystems and substantial economic losses [8]. On the contrary, *M. cordata* grows at a relatively slow rate without ecological harm [9]. Therefore, *M. cordata* and *M. micrantha* are a perfect couple for comparative study which can provide novel insights into the successful establishment of introduced species [10]. Previous comparative studies of *M. cordata* and *M. micrantha* mainly focus on physiological traits [9], chloroplast genome [11], and transcriptome [12], whereas the genomic level analysis lacks. The genome of *M. micrantha* reveals that high photosynthetic efficiency and capability contribute to the successful invasion [13]. Comparative genomics, rather than independent analysis, thus can determine the root causes of functional trait divergence [14,15]. However, the genomic resources of *M. cordata* are lagging far behind *M. micrantha*. The high-quality genome of *M. cordata* is urgently necessary for the forthcoming comparative genomics.

Plants live together with covert endophytes extensively, although many plant genomes have been sequenced and assembled [16]. Therefore, the host DNA may be contaminated with foreign DNA during extraction. The contaminant DNA, if not eliminated, will be sequenced and assembled along with the host DNA [17]. For example, in the genome of *Saccharina japonica* and the domesticated olive, up to 1.5% and 1.3% scaffolds were identified as contaminants, respectively, which were missed in the published genome [18,19]. The most common method to filter contaminants in sequencing is to trim the nontarget sequences in silico based on nucleotide similarity [20], which however risks eliminating sequences from the target organism because of extensive horizontal gene transfer between plant and microbes [21]. In this context, obtaining the aseptic sample represents a fundamental solution for contamination issues. There have been reports that the in vitro aseptic plantlets were used for whole-genome sequencing, such as algae *Spirogloea muscicola*, *Mesotaenium endlicherianum* [22], and poppy family *Macleaya cordata* [23]. For *Mikania* spp., several endophytes are documented, which probably poses the biggest challenge for the axenic culture. Recently, nine fungal endophytes have been isolated from the leaves, twigs, and roots of *M. cordata* [24]. The bacterial endophytes have been identified in *M. micrantha* as well [25]. Additionally, more than 90% of the plantlets contaminated by endophytic bacteria or fungi have been found in micropropagation of *Mikania glomerate* even after surface disinfection [26]. We have also encountered microbial growth during the in vitro culture of *M. cordata*. Further, antibiotics were incorporated into culture media to suppress bacterial growth. Specifically, the bacterial inhibitory effect of two types of β-lactam antibiotics, the widely used cefotaxime (CTX) and the novel timentin (TIM) [27], were assessed on the micropropagation of *M. cordata* in the present study. Since micropropagation enables to efficiently generate genetically faithful clones via tissue culture [28,29], it is reasonable to apply the method to obtain multiple identical plantlets for genome sequencing.

Plant genomes are typically repetitive, which are too complicated to de novo assemble by Illumina short-read sequencing [30]. In this respect, long-read sequencing may exceed the repetitive region in genomes and successfully assemble the contiguous genome [31]. Nevertheless, due to the costly nature of long-read sequencing, it is a convention to perform short-read sequencing in advance. Genome survey sequencing provides a cost-effective strategy to estimate genome size, heterozygosity, and repetitive rate based on short-read data [32]. More importantly, it can also be applied to evaluate the simple sequence repeat (SSR) markers at the genome level. In this work, a micropropagation protocol for producing visibly aseptic *M. cordata* has been developed, and the genome of *M. cordata* has been characterized for the first time. It will help comprehensively for scheming the subsequent long-read sequencing of *M. cordata* genome.

## 2. Results

### 2.1. Micropropagation of M. cordata

**Figure 1:**
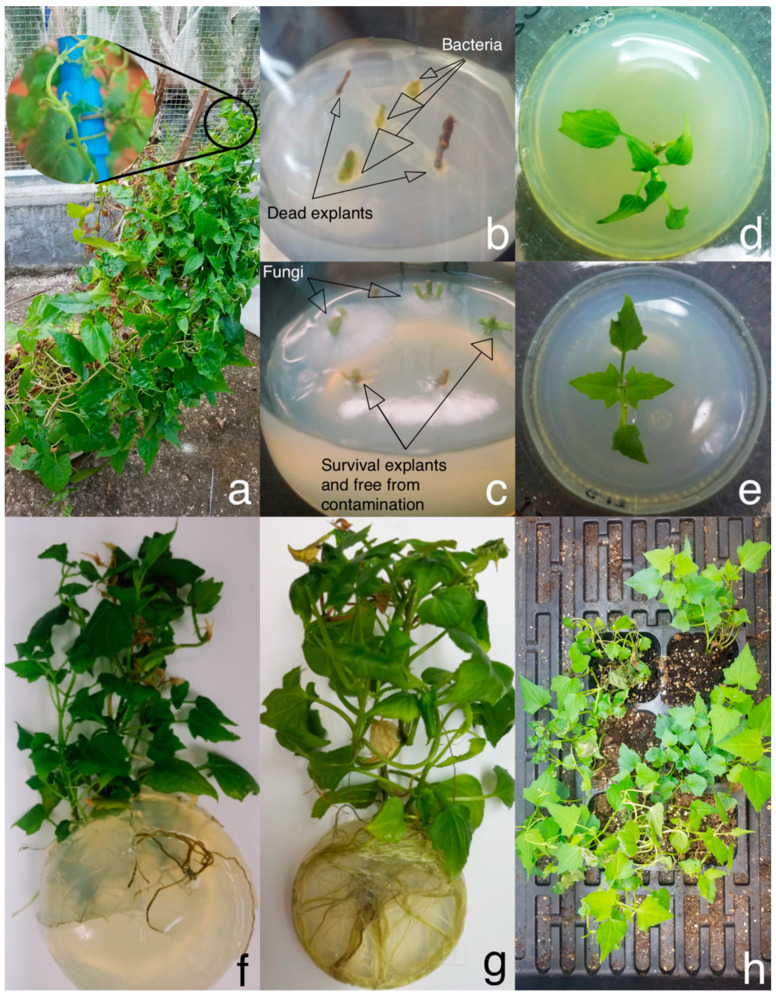
Micropropagation of *M. cordata*. (**a**) A greenhouse-grown *M. cordata* and the juvenile shoots (in the circle) were collected as explant donors. (**b**) On the 6th day, three explants were colonized by bacteria and two explants died in one antibiotic-free medium. (**c**) On the 6th day, two explants were colonized by fungi and two explants were potentially aseptic in one TIM-treated medium. (**d**) The plantlet had sprouted after 6 weeks of cultivation with CTX. (**e**) The plantlet had sprouted after 6 weeks of cultivation with TIM. (**f**) After 8 weeks of cultivation on 1/2 MS media with CTX, the plantlet grew sparse roots with relatively small leaves. (**g**) After 8 weeks of cultivation on 1/2 MS media with TIM, the plantlet grew lush roots with relatively large leaves. (**h**) The robust plantlets were transferred to plastic pots containing soil and sand (1:1) mixture and were able to establish ex vitro after 4 weeks of cultivation. Abbreviation: CTX, cefotaxime; TIM, timentin; MS, Murashige and Skoog.

A total of 51 apical and axillary shoots of *M. cordata* was collected as explant donor of in vitro culture (Figure 1a). Of the antibiotic-free group served by 11 explants, 7 explants were colonized by bacteria while the others were killed by surface disinfection (Figure 1b, Table 1). Fungi colonized 3 of the 20 explants in the CTX-treated group. By contrast, bacteria and fungi colonized four and one of the 20 explants, respectively, in the TIM-treated group (Figure 1c). Initially, five explants were cultured on each Murashige and Skoog (MS) medium. The contaminated explants were discarded once bacteria or fungi appeared, and the remaining explants in the medium were transferred to a fresh medium. In all instances, the microbial colonization became visible within 2 weeks. After 4 weeks of cultivation, four (CTX-treated) and five (TIM-treated) explants were nonviable and also discarded.

Consequently, 11 (CTX-treated) and eight (TIM-treated) explants sprouted after 2 weeks of cultivation and reached up about 2–3 cm in height after 6 weeks in sprouting media (Figure 1d,e, Table 1). The sprouted plantlets were transferred to the rooting media with half of the initial antibiotic concentrations. During the rooting phase, fungi colonized on only one plantlet (TIM-treated) (Table 1). After 8 weeks of cultivation in rooting media, six (CTX-treated) and all the seven (TIM-treated) plantlets rooted (Table 1). The plantlets in the TIM-treated group tended to grow lush roots with large leaves (Figure 1g). In the CTX-treated group, however, the plantlets had sparse roots and small leaves (Figure 1f).

Treatment of 0.3 g·L^−1^ TIM generated well-rooted plantlets; hence, TIM was the only antibiotic used in the subsequent subculture. The axillary shoots of aseptic plantlets from two experimental groups were cut off and subcultured on 1/2 MS media supplemented with TIM. All the plantlets transferring from CTX-containing media to TIM enabled to root. After two rounds of large-scale propagation, more than 300 visibly aseptic plantlets were obtained (Table 1), which met the requirement of DNA extraction. Additionally, the leaves of a batch of aseptic plantlets were harvested for genome survey sequencing. Moreover, most of the ~100 plantlets in long-term maintenance (>300 days) were still visibly aseptic though latent or exogenous microbes grew in a few cultures. Although our goal was to generate aseptic plantlets instead of to establish ex vitro plants, the plantlets were able to establish ex vitro after acclimatization (Figure 1h).

### 2.2. Sequencing, Contigs Assembly, and Preliminary GC Depth Profiling

**Figure 2:**
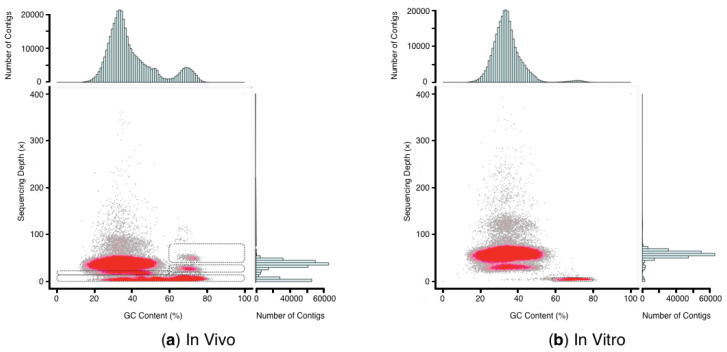
GC content and average sequencing depth distribution of in vivo (**a**) and in vitro (**b**) *M. cordata*. The *x*-axis represents the GC content of contigs while the *y*-axis represents their average depth. Each dot represents an individual contig. The red region indicates high-density portions of the contigs. The extracted regions are indicated by the dashed rectangle. The distribution of contig depth is posited on the right while the distribution of GC content is on the top.

Through Illumina sequencing, a total of 135.4 Gb and 176.5 Gb raw reads were obtained from the in vivo and in vitro culture of *M. cordata* with 89.94% and 92.97% Q30 bases scores, respectively. After removing low-quality reads, 120.1 Gb (in vivo) and 154.3 Gb (in vitro) clean reads were generated. The GC content from the in vivo plant is 37.95%, which is higher than the 36.74% GC content from in vitro plantlets (Table 2). As shown in Figure 2, the GC content and sequencing depth distribution of the >500 bp contigs were plotted, and the red regions represented the high-density portions of the contigs. For in vivo plant, most contigs were concentrated in 20−50% GC content and approximately 25−50× depth region. There were also several high-density stray regions in 20−80% GC content under 15× depth and in 60−100% GC content between 20−80× depth, which may be contributed by the microbial contaminant. For in vitro plantlets, there were three distinct high-density regions. The highest density occurred in the region of 20−50% GC content and around 50−80× depth. Of the same GC content but around 40× depth was another high-density region. The dense stray regions between 57−80% under 10× depth may be caused by the microbial contaminant. The stray regions of in vitro plantlets were shrunk remarkably compared to those of in vivo *M. cordata*.

### 2.3. Identification and Filtration of Genomic Contaminants

Contigs in the stray regions were extracted to confirm their origin. The contigs in four regions from in vivo plant were extracted: 0−100% GC content at 0−14× depth, 0−60% GC content at 14−24× depth, 60−100% GC content at 20−35× depth, and 60−100% GC content at 40−80× depth. The contigs in 57−80% GC content at 0−10× depth from in vitro plantlets were extracted. The top five species distribution of contigs in stray regions was listed in Table 3. The contigs that hit microbes are far less from in vitro plantlets than those from in vivo plant. In the stray regions from in vivo plant, there were overwhelming contigs that hit *Fusarium fujikuroi*, an endophytic fungus capable of causing various types of plant diseases [33]. The most dominant contigs in the high GC content region of both samples hit *Methylobacterium* spp. which were known as the plant growth-promoting bacteria [34]. Of the stray region of 14−24× depth and 0−60% GC content from in vivo plant, contigs were aligned to plant sequences (the top five) followed by a few microbe ones (see Supplementary Table S1). This indicated that the stray region mainly consisted of the low-depth contigs of *M. cordata*.

### 2.4. Genome Assembly and GC Depth Profiling after In Silico Decontamination

**Figure 3:**
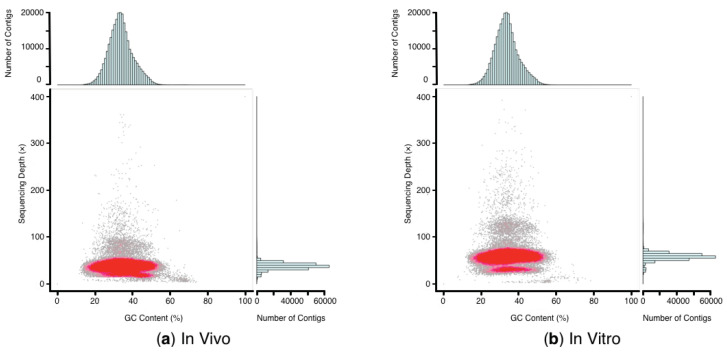
GC content and average sequencing depth distribution of in vivo (**a**) and in vitro (**b**) *M. cordata* after decontamination. The stray regions at high GC content and low depth were both shrunk dramatically compared to Figure 2. (**a**) Due to the relatively low sequencing depth of in vivo plant, the high-density region is more concentrated. However, there remains a small stray region at the low depth and high GC content. (**b**) Due to the relatively high sequencing depth of in vitro plantlets, there are two distinct high-density regions around 76× and 38×, which might be the homologous and heterologous sequences in the genome.

The clean reads and GC content from in vivo plant were greatly decreased by 10.5 Gb and 1.01% after decontamination, respectively. By contrast, only 0.1 Gb clean reads from in vitro plantlets were filtered out, and GC content was slightly decreased by 0.02% (Table 4). A total of 3,555,786 (in vivo) and 3,511,473 (in vitro) >100 bp contigs were assembled, with N50 of 352 bp and 312 bp, respectively (Table 4). After decontamination, the GC depth distribution results showed that the stray regions were dramatically shrunk in both cases (Figure 3). However, there remained a small stray region at low depth with high GC content for in vivo plant, probably caused by the unfiltered microbes with high GC content. Due to the relatively high sequencing depth of in vitro plantlets, there were two distinct high-density regions around 76× and 38×, which might be derived from the homologous and heterologous sequences in the genome.

The >100 bp contigs were further assembled into 1,443,212 (in vivo) and 1,526,309 (in vitro) scaffolds, with N50 of 3087 bp and 2570 bp, respectively (Table 4). The scaffold assembly consisted of 4.33% (in vivo) and 5.02% (in vitro) N bases. The longest contig and scaffold from in vivo plant unusually exceeded those from in vitro plantlets (Table 4). There were 42 and 20 scaffolds from the in vivo plant, which were longer than the longest scaffold from in vitro plantlets, aligned with fungi and bacteria, respectively (see Supplementary Figure S1). To measure the completeness of scaffold assembly, BUSCO analysis was performed showing that 76.08% of 255 core eukaryotic genes were completely identified in the scaffolds of in vitro plantlets, including 68.24% single-copy genes and 7.84% duplicated genes. Additionally, 15.29% and 8.63% were identified as fragmented and missing genes, respectively (see Supplementary Figure S2).

### 2.5. Estimation of Genome Size, Heterozygosity, and Repetitive Rate Based on 17-mer Analysis

**Figure 4:**
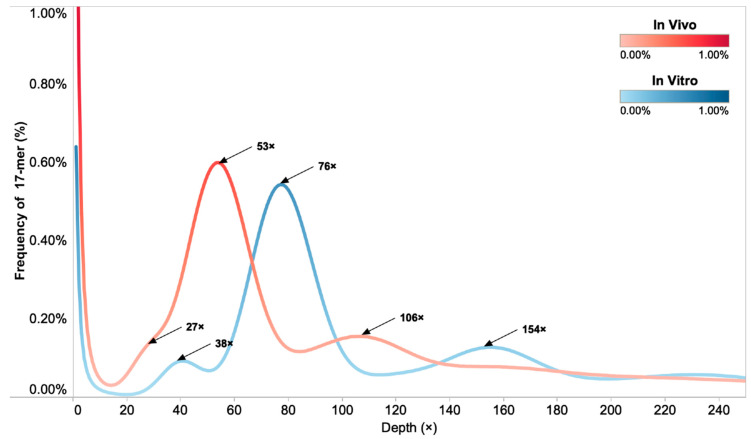
Distribution of 17-mer depth and frequency of in vivo (red line) and in vitro (blue line) *M. cordata*. The highest cut-off frequency is at 1% and the maximum cut-off depth is at 250×. Arrows indicate the half depth, the depth, and the double depth of the main peak, respectively. The main peak represents the overall sequencing depth of the genome. The second-highest peak around the double depth of the main peak represents repetitive sequences. The nonobvious peak at the half depth of the main peak represents the heterozygous peak. The 17-mer distribution suggests that the genome of *M. cordata* is highly repetitive.

The decontaminated clean reads were used for 17-mer frequency analysis for genome size estimation. The number of 17-mer was 97,798,286,149 (in vivo) and 137,835,582,212 (in vitro), respectively (Table 5). The main peak of 17-mer frequency distribution was at 53× (in vivo) and 76× (in vitro) (Figure 4), which represented the overall sequencing depth of the genome. The second and third-highest peaks were separately located at around the double and half depth of the main peak. The estimated genome size was 1,845.25 Mb (in vivo) and 1812.83 Mb (in vitro), respectively. By removing the 17-mer at 1× depth, each size was revised to 1,816.81 Mb (in vivo) and 1801.99 Mb (in vitro). The heterozygosity was calculated as 0.64% (in vivo) and 0.50% (in vitro). The repetitive rate was estimated at 74.72% (in vivo) and 78.35% (in vitro). Overall, results of the 17-mer distribution indicated that the genome of *M. cordata* is highly repetitive.

### 2.6. SSR Identification

**Figure 5:**
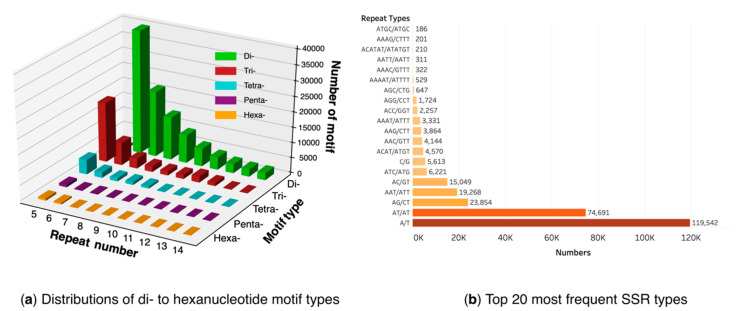
Genome-wide identification of simple sequence repeats (SSRs) in *M. cordata*. (**a**) Generally speaking, the number of simple sequence repeats (SSRs) decreased gradually as the repeat times of motifs increasing; (**b**) The A/T repeat type was the most dominant (119,542), followed by AT/AT (74,691) and AG/CT (23,854).

The scaffolds from in vitro plantlets with a low level of contaminants were utilized for SSR identification to ensure higher reliability. A total of 289,831 SSRs, including 125,155 (43.1%) mono-, 113,695 (39.2%) di-, 38,804 (13.3%) tri-, 9509 (3.3%) tetra-, 1390 (0.48%) penta-, and 1278 (0.44%) hexanucleotide repeats, were identified in 149,441 scaffolds. The mononucleotide repeat was the most abundant type. The di- to hexanucleotide SSRs were grouped according to the repeat times of motifs (from 5–4 repeat times, Figure 5a). Generally, the number of SSR decreased gradually as the motif repeats increasing. However, for trinucleotide SSR with 11 repeats, its number is larger than that of 10 repeats, which was attributed to a large proportion of AAT/ATT in the former. The 20 most frequent SSR types were present in Figure 5b. The A/T repeat type was the most dominant, accounting for 41.2% of all SSRs, followed by AT/AT (25.8%). Several trinucleotides and tetranucleotide repeat types, such as AAT/ATT, ATC/ATG, and ACAT/ATGT, also made up a remarkable proportion.

## 3. Discussion

The initial goal of this study was to characterize the genome of *M. cordata* but was impeded by the endophytic microbes. Endophytes that are ubiquitous in field plants confer benefits to their hosts, such as promoting plant growth and providing protection against biotic and abiotic stresses [35]. However, the endophytic DNA will be mixed into the host genome during extraction and bias the studies of the host plant. Various endophytes have been isolated in *M. cordata* [24], *M. glomerate* [26], and *M. micrantha* [25]. This suggests that endophytes removal becomes an obligatory step in related plant genomic research. To obtain aseptic plantlets, the explants of *M. cordata* were collected and disinfected using the common disinfectant. As observed in in vitro antibiotic-free culture of *M. cordata*, endophytic bacteria did grow on antibiotic-free media, indicating that surface disinfection was ineffective in obtaining the aseptic *M. cordata* as other *Mikania* spp. It has been noted that a >90% contamination rate is not extraordinary in initially established in vitro culture [36].

We performed the in vitro antibiotic-containing culture of *M. cordata* to decontaminate DNA sequences other than *M. cordata*. Micropropagation and tissue culture of *Mikania* species have been documented for *M. glomerate* [26] and *M. micrantha* [37]. Both exogenous and endogenous microbes have been noted as the greatest challenge in the micropropagation of *M. glomerate* [26]. The same case is with *M. cordata*. Eed et al. show that incorporating antibiotics into the in vitro culture may offer a solution to produce aseptic *M. cordata* plantlets [38]. Since fungicides are highly toxic for plants [39], this study only utilized antibiotics to inhibit bacterial growth in micropropagation. The survival rate of explants increased to 65% with 1 g·L^−1^ CTX treatment. Similarly, the survival rate was also increased to 50% under the treatment of 0.6 g·L^−1^ TIM. Among the viable plantlets, the germination rate was above 80% in both treatments. During the rooting phase, the rooting rate with the treatment of 0.5 g·L^−1^ CTX was sharply decreased to 55% in comparison to the 100% rooting rate under the treatment of 0.3 g·L^−1^ TIM. CTX was observed to have toxic effects on the root growth of *M. cordata*. Similar results have been reported for orange [27] and tobacco [40]. It is of note that the rooting ability was recovered after the nonrooting plantlets were transferred to TIM-containing media. Though the latent or exogenous microbes reappeared on a few cultures (~5%), most plantlets were microbial-free during large-scale propagation and maintenance. These results indicated that antibiotics were able to inhibit the growth of bacteria in the long term [41]. Overall, CTX showed more effectiveness at the initial cultivation of *M. cordata* to control endophytes than TIM, but it had an inhibitory effect on rooting. In contrast, TIM did not affect the rooting of *M. cordata*, which has also been demonstrated in tobacco [40] and tomato [42]. As for fungi, they may be eliminated by chance in culture in batches. Previous studies have shown that culturing multiple explants represents an effective way to produce fungal endophyte-free explants for *Mikania* plants [37]. In line with this, a batch of aseptic *M. cordata* was obtained for genome sequencing.

Before sequencing the in vitro plantlets of *M. cordata*, we sampled the greenhouse-grown *M. cordata* for genome survey sequencing and observed 8.7% (10.5 of 120.1 Gb) microbial sequences in clean reads. The contamination in DNA sequencing of *M. cordata* is much higher than that in RNA-Seq of *M. cordata* where only 0.076% unigenes were identified as fungi-origin sequences [12]. Blast results validate that the most dominant contaminant in *M. cordata* is *Fusarium fujikuroi*, a filamentous fungus. Similarly, *F. oxysporum* has also been found in *M. micrantha* rhizosphere but not very abundant [43]. The contaminant DNA will not only take up part of the sequencing resources but also mislead assembly; thus, the in vitro aseptic plantlets were used for genome survey sequencing again. There were only 0.06% (0.1 of 154.3 Gb) clean reads belonging to microbes and the stray region in GC depth distribution became dramatically shrunk, which supported that the micropropagation protocol was a success in reducing the endophytes of *M. cordata*. A few remaining contigs in the stray region were identified as *Methylobacterium* spp. which had also been isolated from the leaves of *M. glomerate* [44] and *M. micrantha* [43]. This study again suggested that the micropropagation media incorporating antibiotics can effectively eliminate the covert endophytes in *M. cordata*. Our protocol may promote the availability of aseptic *M. cordata* in future studies and be potentially applied to other plants whose genome sequencing is interfered with endophytes.

Although *M. cordata* represents a valuable species for comparative study with *M. micrantha*, its genome resources are lacking. The chromosome number of *M. cordata* is 2n = 36 [45], but the genomics information remains absent to date. As plant genomes are generally giant, genome survey by next-generation sequencing offers an affordable way to characterize the genome before conducting costly long-read genome sequencing [46]. Similar strategies have been applied to *Betula platyphylla* [47], *Pistacia vera* [48], and *Rhododendron micranthum* [49]. Genome survey sequencing has been shown that it enables to provide a preliminary understanding of such genomic characteristics as genome size, heterozygosity, and repetitive rate. In this study, we have conducted genome survey sequencing of the in vivo and in vitro culture of *M. cordata*, respectively. By surveying in vitro plantlets, the genomic characteristics may be assessed more accurately. The genome size of *M. cordata* was estimated at 1.80 Gb, which is typical in Asteraceae [50] and comparable with its congeneric *M. micrantha* of 1.86 Gb [13]. However, this size is much smaller than that of *Helianthus annuus* (3.6 Gb) [51]. Several studies have proposed that invasive species with more plasticity tend to have smaller genomes in comparison to their non-invasive relatives [52,53]. Nevertheless, our results highlight that the genome size of invasive species can be larger than that of the native species. We speculate that the large genome of *M. micrantha* may be associated with its CAM photosynthetic characteristics [13], since CAM plant tends to have low stomatal density [54], which is further negatively correlated with genome size [55]. Moreover, the estimated repetitive rate of *M. cordata* (78.35%) is found higher than that of most published genomes of Asteraceae plants, such as *M. micrantha* (73.12%) [13], *Erigeron breviscapus* (54.58%) [56], and *Taraxacum kok-saghyz* (68.56%) [57]. In this study, we observed that the contaminant DNA may mislead the estimate of genomic characterization. Compared with in vitro plantlets, the proportion of heterozygous 17-mer derived from in vivo plant shows an increase in *M. cordata*; but the proportion of repetitive 17-mer demonstrates a decrease. This may cause the heterozygosity overestimated and the repetitive rate underestimated. The contaminants may also lead to 14.82 Mb (0.82% of the whole genome) overestimation of the genome size. Our results are in contrast to the genome survey sequencing of *B. platyphylla* [47], which showed that contaminants brought no interference to genomic characterization.

Furthermore, 1.05 Gb scaffolds were generated from in vitro plantlets, which only covers 58.33% of the estimated genome. This relatively low genome coverage is not unexpected since the high repetitive rate of *M. cordata* may considerably inhibit the assembly of short reads. The longest scaffold generated from the in vivo plant is extremely long (4.29 Mb). Almost all the abnormal long scaffolds from in vivo plant were found aligned with microbial sequences. This finding is in line with the genome survey sequencing of *Hypsibius dujardini* and *B. platyphylla*, where the extremely long but low-abundance assemblies were identified as contaminant sequences [47,58]. In this study, the microbial scaffolds were eliminated from the scaffolds generated from in vitro plantlets. The existence of microbial scaffolds as well as their misleading effects on assembly stresses the necessity to produce in vitro aseptic plantlets of *M. cordata* for future long-read sequencing. It is of note that as much as 76.08% of core eukaryotic genes were found in our assemblies. The percentage is lower than that derived from the high-quality genome of *M. micrantha* (91%) [13] but higher than other assemblies based on short-read sequencing [47,59]. Of scaffold assemblies, the gene coverage was higher than the genome coverage. This might be linked to that hard-to-assemble repetitive regions are gene-poor.

It is conventional to perform simple sequence repeats (SSRs) analyses in genome survey. Due to the low contaminant level of in vitro plantlet, we used its scaffolds for SSRs identification. Additionally, to our knowledge, this study has reported the genomic SSRs of *M. cordata* for the first time. In total, 289,831 SSRs have been identified, which are much more abundant than the 3,602 EST-SSRs identified in *M. micrantha* [60]. The most common motifs of mono-, di, and trinucleotide in *M. cordata* are A/T, AT/AT, and AAT/ATT, respectively. The same pattern has been documented in other plants like jujube, grape, and mulberry [61]. The genomic SSRs developed here would be useful to assess the genetic diversity in *M. cordata* and examine the transferability in *M. micrantha* [62,63].

## 4. Materials and Methods

### 4.1. Plant Materials

A whole plant of wild *M. cordata* was collected from Green Island, Taiwan, China (22°39′40.0′′N, 121°28′59.2′′E) during December 2018 and transplanted in the greenhouse at Sun Yat-sen University, Guangdong, China. After 6 months of planting, the fresh leaves of *M. cordata* were cut and rinsed with distilled water. The leaves were frozen in liquid nitrogen immediately and stored at −80 °C freezer until DNA extraction.

### 4.2. Micropropagation of M. cordata

The apical and axillary shoots were collected from the same *M. cordata* individual cultivated in the greenhouse. After washing under water and trimming the leaves, the juvenile shoots as explant donors were disinfected with 70% ethanol for 2 min, followed by 0.2% sodium hypochlorite for 15 min. Once disinfected, the explants were rinsed 5 times in distilled water and dried using sterile filter paper. The explants were then trimmed into 1 cm segments and cultured on MS media [64] (pH = 5.8) with sucrose (3% *w*/*v*), agar (0.75% *w*/*v*), and 1 mg·L^−1^ benzyl adenine (BA) for sprouting. The antibiotic-containing MS media were either supplemented with 1.0 g·L^−1^ CTX or 0.6 g·L^−1^ TIM. The number of initial explants for culture establishment in each set was listed in Table 1. The cultures were incubated under 16 h light/8 h dark photoperiod at 26 °C for around 6 weeks, after which the sprouting plantlets were selected. The plantlets were subcultured on PGR-free 1/2 MS media containing a corresponding antibiotic reduced to half of the starting concentration for rooting. After 8 weeks of cultivation, the axillary shoots of rooted plantlets from CTX-treated and TIM-treated groups were cut off and subcultured on the 1/2 MS media with 0.3 g·L^−1^ TIM for 8 weeks of large-scale propagation. After that, the axillary shoots of strong plantlets were cut off again and subcultured in the same way for second-round propagation. Large-scale propagation of the aseptic plantlets would be continued until the number of aseptic leaves met the requirement of DNA extraction. Every time the plantlets were transferring to fresh media, the green leaves were harvested and immediately frozen in liquid nitrogen. The leaves were stored at −80 °C freezer until DNA extraction. Around 100 plantlets were successively subcultured for long-term maintenance after sampling. Through the whole period of in vitro culture, the plantlets were discarded once the culture was colonized by bacteria or fungi. A total of 6 of the robust plantlets were acclimatized to the incubation environment for 2 days, and then they were transferred in the soil and sand (1:1) mixture under the same incubation conditions.

### 4.3. DNA Extraction, Library Construction, and Genome Sequencing

The fresh leaves of in vivo mother plant and in vitro plantlets were used for genome survey sequencing, respectively. Briefly, genomic DNA was isolated using the CTAB (Cetyl Trimethyl Ammonium Bromide) method [65]. DNA purity and integrity were monitored on 1% agarose gel electrophoresis, and DNA concentration was measured using Qubit DNA Assay Kit in Qubit 2.0 Fluorometer (Thermo Scientific, Waltham, MA, USA). The genomic DNA of each sample was randomly sheared into short fragments of around 350 bp by M220 Covaris ultrasonicator (Covaris, Woburn, MA, USA). The fragments were subjected to library construction using the Truseq Nano DNA HT Sample Preparation Kit (Illumina, San Diego, CA, USA). The paired-end libraries were sequenced using the Illumina Novaseq 6000 platform (Illumina, San Diego, CA, USA) with read length of 2 × 150 bp by Novogene Co., Ltd. Two sets of raw sequencing reads are available in the Short Read Archive (SRA) database under the accession number of SRR12532532 (the in vitro grown plantlet) and SRR12532533 (the in vivo mother plant).

### 4.4. Contigs Assembly and Preliminary GC Depth Profiling

We utilized FastQC [66] for quality control. To obtain clean reads, for each case below, the entire raw reads were discarded through in-house Perl scripts: (1) the reads containing adapters; (2) the reads with an N ratio higher than 10%; (3) >20% of the entire paired-end reads composed by low-quality base (Phred quality score <5). The clean reads were used for contigs assembling by SOAPdenovo v2.2.7 [67]. Briefly, the clean reads were used to construct a de Bruijn graph with an optimal *k*-mer size of 41. The contigs were then obtained by breaking the connections at low-frequency *k*-mer, repeat boundaries, and unambiguous sequence fragments of the *de Bruijn* graph. The correlation between GC content and average per-base sequencing depth of the >500 bp contigs was plotted using a customized R script.

### 4.5. Identification and Filtration of Genomic Contaminants

Based on the GC depth distribution, the contigs in the distinct stray regions were extracted using a custom-made Perl script. The contigs from stray regions were blasted (Basic Local Alignment Search Tool) against the NCBI (National Center for Biotechnology Information) NT (Nucleotide collection) database with E-value = 1e − 5. The contigs that hit the sequences of microbes were regarded as the contaminating contigs and were subsequently used to build a local contaminant database. After that, the raw reads as queries were blasted against the contaminant database. The raw reads hit, regarded as contaminants, were filtered out. Consequently, the decontaminated raw reads were generated. After trimming the decontaminated raw reads with the same standard as above, the decontaminated clean reads were obtained.

### 4.6. Genome Assembly and GC Depth Profiling after In Silico Decontamination

The decontaminated clean reads were subjected to assemble contigs as above. The GC depth distribution of the >500 bp contigs was plotted again to confirm the contaminants were eliminated. Subsequently, the scaffolds were built by mapping decontaminated clean reads back to the >100 bp contigs using SOAPdenovo v2.2.7. The scaffolds from in vivo plant longer than the longest scaffold from in vitro plantlets were subject to DIAMOND BLASTX [68] against NCBI NR (nonredundant) proteins database with E-value = 1e − 3. The top-hit species distribution of those scaffolds was plotted. The completeness of scaffold assembly from in vitro plantlets was assessed based on 255 core eukaryotic genes by BUSCO (Benchmarking Universal Single-Copy Orthologs) v4 [69].

### 4.7. Estimation of Genome Size, Heterozygosity, and Repetitive Rate Based on 17-mer Analysis

The occurrences of 17-mer were counted for the decontaminated clean reads generated from in vivo plant and in vitro plantlets using Jellyfish 2.2.7 [70]. The distribution of 17-mer depth and frequency was plotted using Tableau. Genome size, heterozygosity, and repetitive rate were estimated using the Genomeye program developed by Novogene Co., Ltd. Genome size was estimated as follows:

(1) $$\mathrm{Genome}\;\mathrm{size}=\frac{\mathrm{No}.\;\mathrm{of}\;17-\mathrm{mers}}{\mathrm{Main}\;\mathrm{peak}\;\mathrm{depth}}$$

Due to sequencing error, there were many low-frequency 17-mers. The proportion of 17-mers with depth = 1 was regarded as the error rate. The genome size was revised via the following formula:

Revised genome size = Genome size × (1 − Error rate)(2)

A small-peak at half of the main peak depth represents the heterozygous peak. The estimated heterozygosity of genome can be calculated as follows:

(3) $$\mathrm{Heterozygosity}=\frac{a_{1/2}\times n_{species}/\left(2\times 17\right)}{n_{species}-a_{1/2}\times n_{species}/2}=\frac{a_{1/2}}{17\times \left(2-a_{1/2}\right)}$$

The n*_species_* stands for the total number of 17-mers species, and a_1/2_ stands for the ratio of heterozygous 17-mers species.

Another small peak at the multiple depth of the main peak represents the repetitive sequences. The 17-mers with the depth 1.8 times higher than the depth of the main peak were regarded as repetitive sequences. The proportion of those 17-mers were predicted to be the repetitive rate in the genome.

### 4.8. SSR Identification

The scaffolds from in vitro plantlets were used to detect SSR using MISA (MIcroSAtellite identification tool) [71]. The minimum number of repeats was set to 10, 6, 5, 5, 5, and 5 for mono-, di-, tri-, tetra-, penta-, and hexanucleotides, respectively. The statistics of SSR motifs were analyzed by Excel 2019 and plotted using the Python matplotlib package and Tableau.

## 5. Conclusions

This is the first report of the whole-genome sequencing of *M. cordata*. Based on 154.2 Gb (85× coverage) of clean reads from in vitro plantlets, the genome size of *M. cordata* is estimated to be 1.80 Gb with 0.50% heterozygosity and 78.35% repetitive rate. A total of 1.05 Gb scaffold was assembled, and 289,831 genomic SSRs were identified. The greenhouse-grown *M. cordata* severely involved microbial contamination, and therefore micropropagation was undertaken with the objectives of obtaining aseptic *M. cordata*. Both CTX and TIM enable to suppress the growth of endophytic bacteria for the in vitro culture of *M. cordata*. Overall, treatment with TIM has performed better in cultivating the robust in vitro plantlets. Our protocol to generate aseptic plantlets by micropropagation and perform genome survey sequencing can be applied to other plants that are heavily contaminated by endophytes. Since the genome of *M. cordata* is highly repetitive, we recommend using long-read sequencing for generating a contiguous genome. To avoid high levels of endophytes infection, the aseptic plantlets of *M. cordata* can be used in future sequencing, which will save considerable sequencing resources as well as achieve a high-quality assembly.

## Figures and Tables

**Table 1:** The effect of antibiotics on the in vitro culture of *M. cordata.*

	Media and PGR (mg·L^−^^1^)	Antibiotic (g·L^−^^1^)	Incubation (Weeks)	No. of Explants/Plantlets	Aseptic Explants/ Plantlets	Survival (>4 weeks)	Sprouting/ Rooting Plantlets
Control	MS + BA 1.0	None	4	11 ^1^	4	0	0
Sprouting	MS + BA 1.0	CTX 1.0	6	20 ^2^	16	13	11
Sprouting	MS + BA 1.0	TIM 0.6	6	20 ^3^	15	10	8
Rooting	1/2 MS	CTX 0.5	8	11	11	11	6
Rooting	1/2 MS	TIM 0.3	8	8 ^4^	7	7	7
Propagation 1st	1/2 MS	TIM 0.3	8	64 ^5^	60	60	60
Propagation 2nd	1/2 MS	TIM 0.3	8	∼350 ^6^	∼330	∼330	∼330

^1^ A total of seven explants were colonized by bacteria. ^2^ A total of four explants were colonized by fungi. ^3^ A total of four explants were colonized by bacteria and one by fungi. ^4^ A total of one plantlet was colonized by fungi. ^5^ A total of three plantlets were colonized by fungi and one by bacteria. ^6^ A total of thirteen plantlets were colonized by fungi and six by bacteria. Abbreviation: PGR, plant growth regulator; MS, Murashige and Skoog Medium; BA, 6-Benzylaminopurine; CTX, cefotaxime; TIM, timentin.

**Table 2:** Statistics for the sequencing data.

	Libraries	Read Length (bases)	Raw Reads (Gb)	Raw Q30 (%)	Clean Reads (Gb)	Clean Q30 (%)	GC Content (%)
In vivo	~350	300	135.4	89.94	120.1	89.26	37.95
In vitro	~350	300	176.6	92.97	154.3	91.81	36.74

**Table 3:** Top five species distribution of contigs in each stray region from NT blast results.

	Region	No. of Contigs	Species	Percentage of Contigs in Stray Region
In vivo	0−14× depth 0−100% GC content	1,041,992	*Helianthus maximiliani*	13.23%
			*Fusarium fujikuroi* ^1^	10.38%
			*Ageratina adenophora*	5.60%
			*Methylobacterium radiotolerans* ^2^	4.15%
			*Methylobacterium extorquens* ^2^	3.86%
			
	14−24× depth 0−60% GC content	152,558	*Helianthus maximiliani*	37.22%
			*Ageratina adenophora*	13.92%
			*Helianthus annuus*	3.85%
			*Solanum lycopersicum*	3.01%
			*Guizotia abyssinica*	2.17%
			
	20−35× depth 60−100% GC content	16,435	*Methylobacterium extorquens* ^2^	15.17%
			*Methylobacterium radiotolerans* ^2^	14.07%
			*Helianthus maximiliani*	6.38%
			*Methylobacterium nodulans* ^2^	3.13%
			*Sphingomonas* sp. ^2^	1.93%
			
	40−80× depth 60−100% GC content	8216	*Helianthus maximiliani*	25.51%
			*Clavibacter michiganensis* ^2^	5.34%
			*Methylobacterium extorquens* ^2^	4.10%
			*Agathis dammara*	3.17%
			*Helianthus annuus*	3.10%
In vitro	0−10× depth 57−80% GC content	7832	*Methylobacterium radiotolerans* ^2^	36.16%
			*Methylobacterium extorquens* ^2^	16.32%
			*Methylobacterium* sp. ^2^	8.96%
			*Brevundimonas subvibrioides* ^2^	5.34%
			*Caulobacter* sp. ^2^	4.34%

^1^ Fungi. ^2^ Bacteria.

**Table 4:** Genome assembly statistics of *M. cordata* after decontamination.

	Clean Reads (Gb)	GC Content (%)	Assembly (>100 bp)	Total Length (bp)	Total Number	Max Length (bp)	N50 (bp)	N Bases (%)
In vivo	109.6	36.94	Contigs	934,276,284	3,555,786	327,948	352	0
			Scaffolds	1,109,673,608	1,443,212	4,285,959	3087	4.33
In vitro	154.2	36.72	Contigs	882,531,142	3,511,473	24,842	312	0
			Scaffolds	1,048,004,173	1,526,309	60,767	2570	5.02

**Table 5:** Statistics of 17–mer analysis for genome size estimation of *M. cordata.*

	Depth	No. of 17-mer	Genome Size (Mb)	Revised Genome Size (Mb)	Heterozygosity (%)	Repetitive Rate (%)
In vivo	53	97,798,286,149	1845.25	1816.81	0.64	74.72
In vitro	76	137,835,582,212	1812.83	1801.99	0.50	78.35

## Supplementary Materials

The following are available online at https://www.mdpi.com/2223-7747/9/12/1665/s1, Figure S1: Top-hit species distribution of top 64 longest scaffolds (all >62 kb) from in vivo *M. cordata*, Figure S2: scaffold assembly evaluation, Table S1: top 100 species distribution of contigs in each stray region from NT blast results.

## Abbreviations

The following abbreviations are used in this manuscript:

BA	6-Benzylaminopurine
BLAST	Basic Local Alignment Search Tool
CAM	Crassulacean Acid Metabolism
CTX	Cefotaxime
MS	Murashige and Skoog
PGR	Plant Growth Regulator
SSR	Simple sequence repeat
TIM	Timentin
