# Supplementary material for: Genome Survey Sequencing of In Vivo Mother Plant and In Vitro Plantlets of Mikania cordata
Source: Plants (Basel). 2020 Nov 27;9(12):1665. doi: 10.3390/plants9121665 (PMC7759884; doi:10.3390/plants9121665)
Supplement: Supplementary file 1 [file plants-09-01665-s001.zip › Figure_S1.pdf]

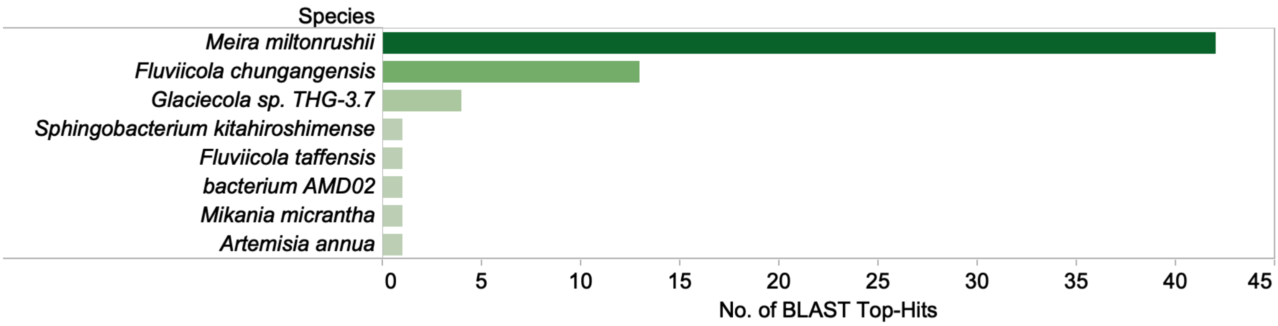

Figure S1. Top-hit species distribution of top 64 longest scaffolds (all >62 kb) from *in vivo M.cordata*. Forty-two and twenty scaffolds from *in vivo* plant that longer than the longest scaffold from *in vitro* plantlets were aligned with fungi and bacteria, respectively. Only two were aligned with plants.
