# Supplementary material for: Genome Survey Sequencing of In Vivo Mother Plant and In Vitro Plantlets of Mikania cordata
Source: Plants (Basel). 2020 Nov 27;9(12):1665. doi: 10.3390/plants9121665 (PMC7759884; doi:10.3390/plants9121665)
Supplement: Supplementary file 1 [file plants-09-01665-s001.zip › Figure_S2.pdf]

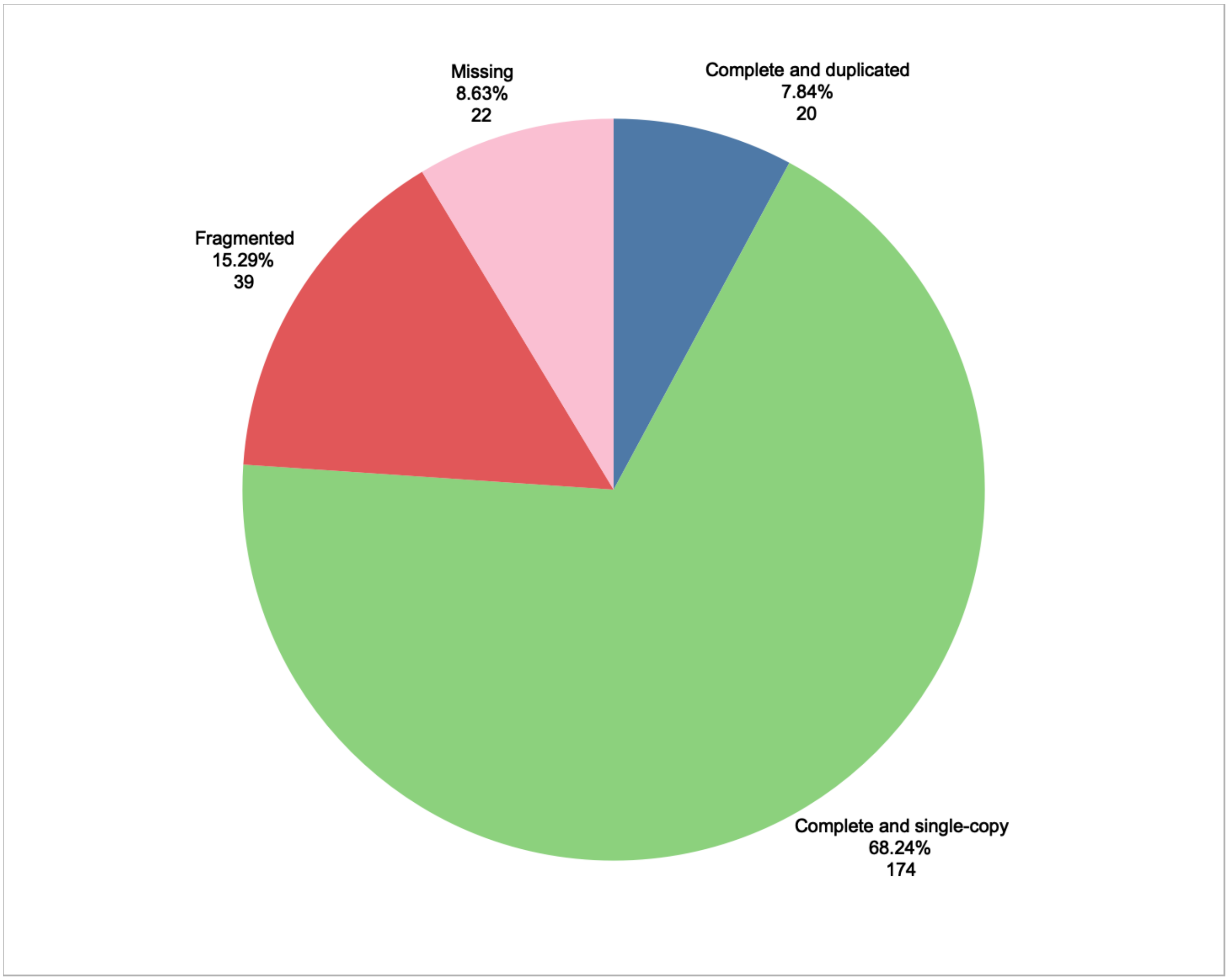

Figure S2. Scaffold assembly evaluation. The BUSCO result of *in vitro M.cordata* from 255 core eukaryotic genes demonstrated in the pie chart. The percentage and number below label indicate the proportion and count of the identified genes for each category. The result showed that 76.08% of 255 core eukaryotic genes were completely identified in the scaffolds.
